# Cardiac resynchronization therapy with a defibrillator in non‐ischemic and ischemic patients for primary and secondary prevention of sudden cardiac death: Analysis of the Japan cardiac device treatment registry database

**DOI:** 10.1002/joa3.12916

**Published:** 2023-08-24

**Authors:** Hisashi Yokoshiki, Akihiko Shimizu, Takeshi Mitsuhashi, Kohei Ishibashi, Tomoyuki Kabutoya, Yasuhiro Yoshiga, Yusuke Kondo, Haruhiko Abe, Wataru Shimizu, Members of the Implantable Cardioverter‐Defibrillator (ICD) Committee of the Japanese Heart Rhythm Society

**Affiliations:** ^1^ Department of Cardiovascular Medicine Sapporo City General Hospital Sapporo Japan; ^2^ UBE Kohsan Central Hospital Ube Japan; ^3^ Department of Cardiovascular Medicine Hoshi General Hospital Koriyama Japan; ^4^ Department of Cardiovascular Medicine National Cerebral and Cardiovascular Center Suita Japan; ^5^ Division of Cardiovascular Medicine, Department of Medicine Jichi Medical University School of Medicine Shimotsuke Japan; ^6^ Division of Cardiology, Department of Medicine and Clinical Science Yamaguchi University Graduate School of Medicine Yamaguchi Japan; ^7^ Department of Cardiovascular Medicine Chiba University Graduate School of Medicine Chiba Japan; ^8^ Department of Heart Rhythm Management University of Occupational and Environmental Health Kitakyushu Japan; ^9^ Department of Cardiovascular Medicine Nippon Medical School Bunkyo City Japan

**Keywords:** cardiac resynchronization therapy with a defibrillator (CRT‐D), implantable cardioverter‐defibrillator (ICD), non‐ischemic, primary prevention, ventricular fibrillation (VF), ventricular tachycardia (VT)

## Abstract

**Background:**

Panoramic studies in patients with cardiac resynchronization therapy with a defibrillator (CRT‐D) focusing on the etiology and indication are scarce. Besides, a controversy exists regarding requirement of a defibrillator in non‐ischemic patients for primary prevention with CRT.

**Methods:**

Annual trends of de novo CRT‐D implantations from 2011 to 2020 and outcomes of those between January 2011 and August 2015 were analyzed from the Japan cardiac device treatment registry (JCDTR) and New JCDTR database.

**Results:**

From 2011 to 2020, 8062 CRT‐D recipients were registered, whose dominant indication was primary prevention of sudden cardiac death with a steady rate of about 70%. There was no significant temporal change of the proportion of non‐ischemic patients being about 70% and 65% for primary and secondary prevention, respectively. Non‐ischemic patients for primary prevention were associated with increased odds of appropriate ICD therapy [adjusted hazard ratio (aHR): 1.66; 95% confidence interval (CI): 1.01–2.75; *p* = .047] and reduced odds of any death (aHR: 0.66; 95% CI: 0.44–0.99; *p* = .046) as compared to ischemic patients.

**Conclusions:**

Proportion of non‐ischemic etiology was much higher than that of ischemic one in the CRT‐D cohort. Based on the higher odds of appropriate ICD therapy, non‐ischemic patients for primary prevention appear to be prudently selected in Japan.

## Graphical abstract

Proportion of non‐ischemic etiology was much higher than that of ischemic one in the CRT‐D cohort being about 70% and 65% for primary and secondary prevention, respectively. Non‐ischemic CRT‐D recipients for primary prevention of sudden cardiac death had a higher rate of appropriate ICD therapy and a lower rate of all‐cause death as compared to ischemic CRT‐D recipients in the JCDTR database.

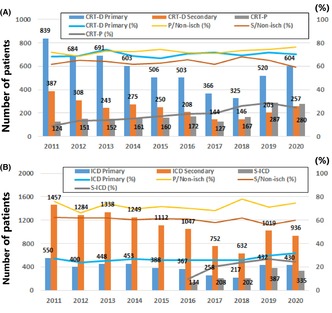

## 1 INTRODUCTION

Cardiac resynchronization therapy (CRT), in combination with guideline‐directed medical therapy (GDMT), is an essential therapy for symptomatic heart failure with reduced left ventricular ejection fraction (LVEF) and prolonged QRS duration of left bundle branch block‐type morphology in both ischemic and non‐ischemic etiologies.^1^, ^2^, ^3^, ^4^ There is no doubt for cardiologists to apply CRT with a defibrillator (CRT‐D) in such patients who had sustained ventricular tachycardia (VT) or ventricular fibrillation (VF). On the other hand, the application and outcome may differ between ischemic and non‐ischemic patients for primary prevention of sudden cardiac death, because reverse structural left ventricular remodeling occurred more favorably in non‐ischemic cardiomyopathy, whereas a defibrillator back‐up appeared to be more effective in ischemic cardiomyopathy.^5^, ^6^, ^7^, ^8^, ^9^ In this regard, we aimed to perform a panoramic study to clarify the temporal trend, characteristic and outcome of non‐ischemic versus ischemic CRT‐D recipients in primary and secondary prevention cohorts by analyzing the Japan Cardiac Device Treatment Registry (JCDTR) database.

## 2 METHODS

### 2.1 Study population

The JCDTR was established in 2006 by the Japanese Heart Rhythm Society (JHRS) for a survey of actual conditions in patients undergoing de novo implantation of cardiac implantable electronic devices (CIEDs) including implantable cardioverter‐defibrillator (ICD)/cardiac resynchronization therapy with a defibrillator (CRT‐D)/cardiac resynchronization therapy with a pacemaker (CRT‐P) (https://center6.umin.ac.jp/islet/icd/index.htm/ accessed on February 9, 2023).^10^, ^11^, ^12^ A new system, called New JCDTR, started on January 2019, in which data of patients at the implantation date after January 2018 are encouraged to register (https://membnew.jhrs.or.jp/newjcdtr/ accessed on February 9, 2023).^13^ The protocol for this research project has been approved by a suitably constituted Ethics Committee at each institution and it conforms to the provisions of the Declaration of Helsinki.

For the analysis of the annual trend, the data of implantation date from 2011 to 2017 and those from 2018 to 2020 were obtained from the JCDTR and New JCDTR, respectively. We also analyzed the characteristics and outcome of consecutive 906 CRT‐D patients whose implantation date was from January 2011 to August 2015 and whose follow‐up data were available as of September 16, 2015.

### 2.2 Device programming

In general, device programming was as follows. The VF zone detected ventricular events faster than 185–200 beats/min with at least one train of anti‐tachycardia pacing (ATP) before shock, and the VT zone detected ventricular events faster than 150–170 beats/min with at least three trains of ATP before shock. After the multicenter automatic defibrillator implantation trial – reduce inappropriate therapy (MADIT‐RIT) trial was published in 2012,^14^ the VF zone ≧ 200–250 beats/min with ATP plus shock and VT zone ≧ 170 beats/min with delayed therapy (a 60 s delay) or only monitoring were recommended. The discrimination algorithms were used at the physician's discretion.

### 2.3 Outcome

The analyzed events were (a) death from any cause, (b) appropriate and (c) inappropriate ICD therapy. Appropriate ICD therapy was defined as an anti‐tachycardia pacing or shock for tachyarrhythmia determined to be either ventricular tachycardia (VT) or ventricular fibrillation (VF). The diagnosis was made by attending physicians.

### 2.4 Statistical analysis

All data are expressed as mean ± SD. Simple between‐group analysis was conducted using Student's *t*‐test. Categorical variables were compared using Fisher's exact test. Kaplan‐Meier curves were constructed to estimate event‐free outcomes in the study groups with comparison using the log‐rank test. Hazard ratios were computed with a multivariate Cox proportional‐hazards regression model after adjusting for confounding factors including age, gender, LVEF, and NYHA class. All reliable variables associated with appropriate ICD therapy (*p* < .10) were entered into a multivariable model using forward stepwise regression analysis. Differences with *p* < .05 were considered significant. Statview version 5.0 for Windows (SAS Institute Inc.) or R software ver.3.6.3 (https://www.r‐project.org/) was used for all statistical analyses.

## 3 RESULTS

### 3.1 Annual trends in CRT‐D, CRT‐P, and ICD implantations

The database had data of 8062 patients, 1781 patients, and 14,769 patients who underwent de novo implantations of CRT‐D, CRT‐P, and ICD (including S‐ICD after February, 2016) from January 2011 to December 2020.

**FIGURE 1:**
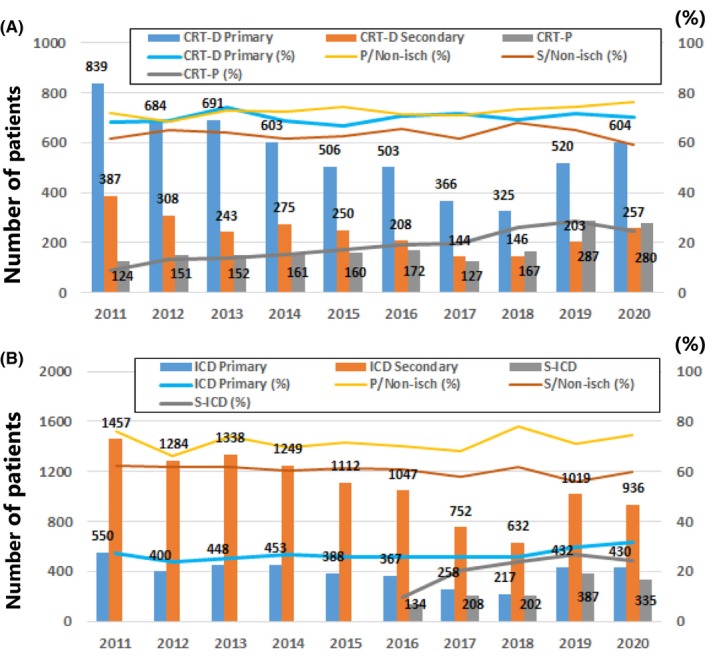
Annual trend in CRT‐D/ICD implantation stratified by indication. The absolute number of primary and secondary prevention of sudden cardiac death is given by blue bar and orange bar for (A) CRT‐D and (B) ICD. The number of CRT‐P (A) and S‐ICD (B) implantation is also shown. Blue line indicates the proportion of primary prevention. Yellow and orange lines indicate the proportion of the primary and secondary prevention in non‐ischemic cardiomyopathy, respectively. Gray line indicates the proportion of CRT‐P implantation among all CRT devices (A) and S‐ICD implantation among all ICD devices (B). CRT‐D, cardiac resynchronization therapy with a defibrillator; CRT‐P, cardiac resynchronization therapy with a pacemaker; ICD, implantable cardioverter‐defibrillator; Non‐isch, non‐ischemic cardiomyopathy; Primary, primary prevention of sudden cardiac death; Secondary, secondary prevention of sudden cardiac death; S‐ICD, subcutaneous ICD.

Proportion of primary prevention CRT‐D implantations was 70.1 ± 2.1% overall, and there was no significant difference with regard to the temporal trend (*p* = .077). Proportion of the non‐ischemic etiology was 72.8 ± 2.1% in the primary prevention group and 63.4 ± 2.5% in the secondary prevention group. Similarly, the temporal trend was not apparent (*p* = .21 for primary prevention; *p* = .80 for secondary prevention). Difference in the etiology of CRT‐D implantations between primary and secondary prevention groups was significant (*p* < .0001). There was a significant increase in the proportion of CRT‐P implantations (*p* < .0001), which was 24.5% of all CRT implantations in 2020 (Figure 1A).

Proportion of primary prevention ICD implantations was 26.7 ± 2.3% overall and there was a significant increase especially in recent years (*p* < .0001). Proportion of the non‐ischemic etiology was 72.0 ± 3.6% in the primary prevention group and 60.4 ± 2.0% in the secondary prevention group. There was a slight increase in the proportion of the non‐ischemic etiology in the primary prevention group (*p* = .012), but no apparent change in that of the secondary prevention group (*p* = .091). Difference in the etiology of ICD implantations between primary and secondary prevention groups was significant (*p* < .0001). There was a significant increase in proportion of S‐ICD implantations (*p* < .0001), which was 24.5% of all ICD implantations in 2020 (Figure 1B).

### 3.2 Patient characteristics

Among 906 CRT‐D patients from January 2011 to August 2015, 620 patients (68%) and 286 patients (32%) were implanted for primary and secondary prevention of sudden cardiac death. Younger age, less male predominance, higher history of non‐sustained VT, and less comorbidities (e.g., diabetes mellitus, hypertension, and dyslipidemia) were observed in non‐ischemic patients of the primary prevention group as compared to ischemic patients. In contrast, with regard to age and history of non‐sustained VT, there was no difference between non‐ischemic and ischemic patients of the secondary prevention group. There was no difference in LVEF, QRS duration, presence of atrial fibrillation and the level of BNP between the two etiologies of the primary and secondary prevention groups. Distribution of the NYHA class was not different between the two etiologies of the primary prevention group, but less severe in the non‐ischemic patients of the secondary prevention group. The level of hemoglobin was higher and that of creatinine was lower in the non‐ischemic than ischemic patients of both the primary and secondary prevention groups (Table 1).

**TABLE 1:** Characteristics of the patients.

	Primary prevention (*n* = 620)			Secondary prevention (*n* = 286)		
	Non‐ischemic (*n* = 447)	Ischemic (*n* = 173)	*p* Value	Non‐ischemic (*n* = 182)	Ischemic (*n* = 104)	*p* Value
Age (years)	65.5 ± 11.5	70.1 ± 9.7	<.0001	65.3 ± 11.8	67.2 ± 9.7	.17
Male	317 (70.7)	159 (91.9)	<.0001	122 (67.0)	91 (87.5)	.0001
LVEF (%)	26.5 ± 9.5	26.5 ± 7.5	.99	29.3 ± 11.4	28.5 ± 9.3	.54
*NYHA class*			.44			<.0001
I	7 (1.6)	1 (0.6)		20 (11.0)	9 (8.7)	
II	117 (26.2)	48 (27.7)		70 (38.5)	32 (30.8)	
III	281 (62.9)	102 (59.0)		78 (42.9)	48 (46.2)	
IV	42 (9.4)	22 (12.7)		14 (7.7)	15 (14.4)	
Heart rate (/min)	71.6 ± 17.9	69.8 ± 15.3	.23	67.6 ± 15.4	70.3 ± 16.9	.17
QRS duration (ms)	153.2 ± 31.7	154.2 ± 28.1	.70	152.5 ± 35.0	145.9 ± 31.4	.11
QT interval (ms)	455.2 ± 55.5	455.5 ± 55.3	.95	460.7 ± 55.6	456.8 ± 54.9	.57
Cardiothoracic ratio (%)	59.4 ± 6.6	58.2 ± 6.0	.037	59.3 ± 6.9	57.2 ± 6.8	.016
*Atrial lead*			.09			.0003
Absent	73 (16.3)	19 (11.0)		25 (13.7)	1 (1.0)	
Present	374 (83.7)	154 (89.0)		157 (86.3)	103 (99.0)	
NSVT^a^	142 (71.4)	37 (52.9)	.005	36 (63.2)	24 (64.9)	.86
AF	60 (13.4)	22 (12.7)	.82	17 (9.3)	11 (10.6)	.74
Diabetes mellitus	111 (24.8)	84 (48.6)	<.0001	41 (22.5)	45 (43.3)	.0002
Hypertension	148 (33.1)	94 (54.3)	<.0001	68 (37.4)	55 (52.9)	.011
Dyslipidemia	98 (21.9)	88 (50.9)	<.0001	39 (21.4)	46 (44.2)	<.0001
Hyperuricemia	87 (19.5)	40 (23.1)	.31	27 (14.8)	18 (17.3)	.58
Cerebral infarction	28 (6.3)	16 (9.2)	.19	9 (4.9)	8 (7.7)	.34
Peripheral artery disease	9 (2.0)	10 (5.8)	.015	2 (1.1)	6 (5.8)	.021
BNP (pg/mL)^b^	806 ± 1461	715 ± 862	.48	560 ± 661	1105 ± 4180	.12
Hemoglobin (g/dL)^c^	12.9 ± 2.0	12.3 ± 2.2	.0003	13.0 ± 1.9	12.2 ± 2.1	.0007
Creatinine (mg/dL)^d^	1.38 ± 1.49	1.74 ± 1.42	.007	1.1 ± 1.48	1.42 ± 1.59	.001

*Note*: Values are means ± SD, or number (%).

Abbreviations: AF, atrial fibrillation; LVEF, left ventricular ejection fraction; NSVT: non‐sustained ventricular tachycardia.

^a^ Information about the presence or absence of NSVT was available in 199 non‐ischemic and 70 ischemic patients with primary prevention and 57 non‐ischemic and 37 ischemic patients with secondary prevention.

^b^ The value of BNP was missed in 45 non‐ischemic and 33 ischemic patients with primary prevention and 22 non‐ischemic and 16 ischemic patients with secondary prevention.

^c^ The value of hemoglobin was missed in two non‐ischemic and one ischemic patients with primary prevention and three non‐ischemic and three ischemic patients with secondary prevention.

^d^ The value of creatinine was missed in five non‐ischemic and one ischemic patients with primary prevention and six non‐ischemic and three ischemic patients with secondary prevention.

Regarding pharmacological therapy, there was no difference in the use of beta blocker, angiotensin converting enzyme inhibitor (ACEI) or angiotensin II receptor blocker (ARB), mineralocorticoid receptor antagonist, and class III anti‐arrhythmic drug between the non‐ischemic and ischemic patients in both primary and secondary prevention groups. The rate of having nitrates, statins, and antiplatelet drugs was lower in the non‐ischemic than ischemic patients of the primary and secondary prevention groups (Table 2).

**TABLE 2:** Pharmacological therapy.

	Primary prevention (*n* = 620)			Secondary prevention (*n* = 286)		
	Non‐ischemic (*n* = 447)	Ischemic (*n* = 173)	*p* Value	Non‐ischemic (*n* = 182)	Ischemic (*n* = 104)	*p* Value
Ia	3 (0.7)	2 (1.2)	.54	5 (2.7)	1 (1.0)	.31
Ib	9 (2.0)	5 (2.9)	.51	5 (2.7)	7 (6.7)	.11
Ic	2 (0.4)	3 (1.7)	.11	1 (0.5)	0 (0.0)	.45
Beta blockers	349 (78.1)	128 (74.0)	.28	135 (74.2)	79 (76.0)	.74
III	135 (30.2)	64 (37.0)	.10	117 (64.3)	72 (69.2)	.40
Ca^2+^ antagonists	32 (7.2)	21 (12.1)	.047	14 (7.7)	14 (13.5)	.11
Digitalis	63 (14.1)	20 (11.6)	.41	22 (12.1)	6 (5.8)	.08
Diuretics	355 (79.4)	144 (83.2)	.28	131 (72.0)	73 (70.2)	.75
ACEI/ARB	300 (67.1)	113 (65.3)	.67	125 (68.7)	63 (60.6)	.16
MRA	204 (45.6)	73 (42.2)	.44	64 (35.2)	35 (33.7)	.80
Nitrates	31 (6.9)	42 (24.3)	<.0001	7 (3.8)	16 (15.4)	.0006
Statins	95 (21.3)	97 (56.1)	<.0001	49 (26.9)	54 (51.9)	<.0001
Oral anticoagulants	243 (54.4)	82 (47.4)	.12	102 (56.0)	47 (45.2)	.08
Antiplatelet drugs	94 (21.0)	147 (85.0)	<.0001	34 (18.7)	87 (83.7)	<.0001

*Note*: Data are given as numbers (%). Ia, Ib, Ic, and III indicate the class Ia, Ib, Ic, and III antiarrhythmic drug, respectively.

Abbreviations: ACEI, angiotensin converting enzyme inhibitor; ARB, angiotensin II receptor blocker; MRA: mineralocorticoid receptor antagonist.

### 3.3 Outcomes

Appropriate ICD therapy occurred more frequently in 85 of 447 non‐ischemic patients (19.0%) versus 20 of 173 ischemic patients (11.6%) of the primary prevention group during a mean follow‐up period of 21 ± 12 months. The rate was 11.9% at 1 year and 21.2% at 2 year in the non‐ischemic patients, and 6.8% at 1 year and 13.6% at 2 year in the ischemic patients (*p* = .049). There was no significant difference with regard to the incidence of appropriate ICD therapy between non‐ischemic (53 of 182, 29.1%) and ischemic (24 of 104, 23.1%) patients of the secondary prevention group. The rate was 23.6% at 1 year and 33.3% at 2 year in the non‐ischemic patients, and 14.0% at 1 year and 29.0% at 2 year in the ischemic patients (*p* = .24).

Inappropriate ICD therapy similarly occurred in 29 of 447 non‐ischemic patients (6.4%) and 8 of 173 ischemic patients (4.6%) of the primary prevention group. The rate was 4.6% at 1 year and 7.4% at 2 year in the non‐ischemic patients, and 2.6% at 1 year and 3.6% at 2 year in the ischemic patients (*p* = .51). In the secondary prevention group, the incidence of inappropriate ICD therapy was higher in ischemic (9 of 104, 8.7%) than non‐ischemic (6 of 182, 3.3%) patients. The rate was 6.1% at 1 year and 7.8% at 2 year in the ischemic patients, and 3.0% at 1 year and 3.0% at 2 year in the non‐ischemic patients (*p* = .041).

Death from any cause occurred less frequently in 72 of 447 non‐ischemic patients (16.1%) versus 42 of 173 ischemic patients (24.2%) of the primary prevention group. The rate was 9.9% at 1 year and 16.1% at 2 year in the non‐ischemic patients, and 13.3% at 1 year and 23.6% at 2 year in the ischemic patients (*p* = .0088). There was no significant difference with regard to the incidence of death between non‐ischemic (35 of 182, 19.2%) and ischemic (18 of 104, 17.3%) patients of the secondary prevention group. The rate was 12.6% at 1 year and 20.8% at 2 year in the non‐ischemic patients, and 10.6% at 1 year and 20.8% at 2 year in the ischemic patients (*p* = .72).

An adjusted hazard ratio (HR) for appropriate ICD therapy and death from any cause was 1.66 [95% confidence interval (CI): 1.01–2.75; *p* = .047] and 0.66 (95% CI: 0.44–0.99; *p* = .046) respectively, in non‐ischemic patients when compared with ischemic patients of primary prevention group (Table 3).

**TABLE 3:** Hazard ratios for events in non‐ischemic versus ischemic patients implanted with a CRT‐D.

	Primary prevention			Secondary prevention		
Events	Hazard ratio	95% CI	*p* Value	Hazard ratio	95% CI	*p* Value
*Appropriate ICD therapy*						
Unadjusted analysis	1.62	0.99–2.64	.052	1.33	0.82–2.16	.24
Adjusted analysis	1.66	1.01–2.75	.047	1.51	0.92–2.49	.10
*Inappropriate ICD therapy*						
Unadjusted analysis	1.30	0.59–2.85	.51	0.35	0.12–1.00	.051
Adjusted analysis	1.17	0.52–2.65	.69	0.34	0.11–1.02	.056
*Death from any cause*						
Unadjusted analysis	0.60	0.41–0.88	.0096	1.10	0.62–1.95	.72
Adjusted analysis	0.66	0.44–0.99	.046	1.34	0.74–2.43	.32

*Note*: Models were adjusted for the following covariates: age at enrollment, gender, LVEF, and NYHA class.

Abbreviations: CI, confidence interval; ICD, implantable cardioverter‐defibrillator; LVEF: left ventricular ejection fraction.

### 3.4 Factors associated with appropriate ICD therapy

Univariate variables (*p* < .10) associated with appropriate ICD therapy were secondary prevention, non‐ischemic etiology, LVEF > 35%, NYHA class I–II, QRS width < 150 ms, performing electrophysiological study (EPS), class III antiarrhythmic drugs, and absence of ACEI/ARB. In multivariate analysis, significant variables were secondary prevention (HR: 1.84; 95% CI: 1.36–2.48; *p* < .0001), non‐ischemic etiology (HR: 1.49; 95% CI: 1.06–2.11; *p* = .022), LVEF > 35% (HR: 1.50; 95% CI: 1.04–2.17; *p* = .031), QRS width < 150 ms (HR: 1.35; 95% CI: 1.01–1.81; *p* = .045), and absence of ACEI/ARB (HR: 1.51; 95% CI: 1.12–2.03; *p* = .0065).

### 3.5 Induction rate of VT/VF by Electrophysiologic study and ICD therapy

Among 125 patients (of 906 CRT‐D patients; 14%) who underwent electrophysiologic study (EPS), sustained VT or VF (VT/VF) was induced in 71 patients (57%). The induction rate was higher in patients with secondary prevention compared to primary prevention (45%, 29 of 64 primary prevention vs. 69%, 42 of 61 secondary prevention patients, *p* = .0079). On the other hand, there was no significant difference with regard to the induction rate between non‐ischemic (53%, 44 of 83) and ischemic (64%, 27 of 42) patients. Appropriate ICD therapy occurred in 35% of patients (25 of 71) with VT/VF induction versus 20% of patients (11 of 54) without VT/VF induction (HR: 1.94; 95% CI: 0.95–3.95; *p* = .067, on univariate Cox regression analysis). Inappropriate ICD therapy occurred in 8% of patients (6 of 71) with VT/VF induction versus 2% of patients (1 of 54) without VT/VF induction (HR: 5.10; 95% CI: 0.61–42.5; *p* = .13, on univariate Cox regression analysis). The detail is summarized in Table S1.

## 4 DISCUSSION

Reverse remodeling in response to CRT occurred more favorably, whereas an ICD back‐up appeared to be less necessary in non‐ischemic versus ischemic patients in several studies from the United States of America and European countries.^1^ More recently, non‐ischemic heart failure patients with LVEF ≦ 35% did not have any survival benefit from prophylactic ICDs and the effect of ICD implantation was independent of CRT status.^15^ On the other hand, the present study demonstrated that appropriate ICD therapy occurred more frequently in the primary prevention CRT‐D patients of non‐ischemic etiology with the lower rate of death from any cause than the patients of ischemic etiology. In this regard, the application of CRT‐D for primary prevention of sudden cardiac death in systolic heart failure patients appears to be appropriately performed in Japan.

Proportion of the non‐ischemic etiology of primary prevention CRT‐D implantations was about 70% in Japan, which was higher than that in the US and Europe.^1^ Despite an increasing trend of non‐ischemic heart failure patients of aged ≥75 in Japan,^13^ there was no temporal change with regard to the proportion of the non‐ischemic CRT‐D patients for primary prevention. In contrast, the proportion of CRT‐P implantations among all CRT devices is increasing (Figure 1A), which is likely due to the increased trend of CRT‐P implantations in patients of aged ≥75. This was reported in our previous study.^13^ Our preference of CRT‐P to CRT‐D for the aged population may reasonably identify non‐ischemic patients at risk of VT/VF,^12^, ^16^ because there was no significant difference in all‐cause mortality between patients with CRT‐D and CRT‐P.^16^ These findings are reasoned by an observation that proportion of sudden death diminished progressively with advancing age.^17^ Additionally, an optimal age cutoff for ICD implantation for reducing all‐cause mortality was 70 years old or less in the DANISH study.^18^

We previously reported that the inducibility of VT/VF by programmed ventricular stimulation was not a predictor of appropriate ICD therapy in heart failure patients with reduced LVEF (≦35%),^19^ which is in agreement with sub‐analyses of the MUSST^20^ and MADIT‐II.^21^ In the present study, we had performed EPS in 125 of 906 CRT‐D recipients (14%), who had a higher risk of appropriate ICD therapy with a univariate analysis. On the other hand, the multivariate analysis did not identify performing EPS as the predictor, whereas secondary prevention, non‐ischemic etiology, LVEF >35%, QRS width < 150 ms and no use of ACEI/ARB were significant risk factors for appropriate ICD therapy. These findings reinforce the evidence that EPS is not a suitable method to identify heart failure patients with reduced LVEF (≦35%) who are at risk of malignant ventricular arrhythmias.

The severity of heart failure was not always correlated to sudden cardiac death and the defibrillator benefit,^22^, ^23^ but was related to the mortality. The benefit of ICDs had an inverse correlation with the advancing NYHA class in ICD or CRT‐D patients.^24^, ^25^ Similarly, there was an inverse correlation between LVEF and the risk of life‐threatening ventricular arrhythmias, possibly contributing to the attenuation in the antiarrhythmic properties of CRT in mild heart failure patients with LVEF >30%.^26^ Additionally, patients with LVEF >35% are not usually candidates for CRT and those implanted with CRT‐D may have any risks of malignant ventricular arrhythmias, which was judged by attending physicians. These observations and speculation may explain why LVEF >35% was one of significant predictors for appropriate ICD therapy.

### 4.1 Study limitations

There are several limitations to be considered in this study. First, ischemic patients had more comorbidities such as diabetes mellitus, hypertension, and dyslipidemia, which is inevitable in nature. These variables were not adjusted for the outcome analyses in Table 3 because of a high degree of collinearity. Second, device programming for ventricular arrhythmias was left on the discretion of attending physicians. However, participants in this cohort study have a certificate of Heart Failure Treatment with ICD and Cardiac Pacing endowed by the JHRS and Japanese Heart Failure Society (JHFS) and would be familiar with contemporary programming. Third, information regarding the presence or absence of non‐sustained VT and atrial fibrillation, which is likely to affect outcomes,^27^ is not mandatory information for the registration in the JCDTR. Fourth, the JCDTR requested to register appropriate ICD therapy as an event, but it did not discriminate between anti‐tachycardia pacing and shock.

## 5 CONCLUSIONS

Non‐ischemic CRT‐D recipients for primary prevention of sudden cardiac death had a higher rate of appropriate ICD therapy and a lower rate of all‐cause death as compared to ischemic CRT‐D recipients in the JCDTR database. With regard to CRT‐D recipients for secondary prevention, there was no difference in those outcomes between the non‐ischemic and ischemic etiology. We appear to identify heart failure patients with prolonged QRS duration at risk of malignant ventricular arrhythmias appropriately, especially in those with non‐ischemic etiology, which is predominant with a rate of about 70% in Japan.

## CONFLICT OF INTEREST STATEMENT

All authors declare no conflict of interest related to this study.

## ETHICS STATEMENT

The JCDTR and New JCDTR were approved by the Ethics Committee of Sapporo City General Hospital on May 16, 2018 (Approval No.:H30‐057‐455).

## CONSENT STATEMENT

Patient consent has been obtained in an opt‐out manner in Sapporo City General Hospital.

## CLINICAL TRIAL REGISTRATION

There is no clinical trial registration number regarding the JCDTR and New JCDTR.

## Supporting information


Table S1.
