# Supplementary material for: Cardiac resynchronization therapy with a defibrillator in non‐ischemic and ischemic patients for primary and secondary prevention of sudden cardiac death: Analysis of the Japan cardiac device treatment registry database
Source: J Arrhythm. 2023 Aug 24;39(5):757–65. doi: 10.1002/joa3.12916 (PMC10549811; doi:10.1002/joa3.12916)
Supplement: Supplementary file 1 — Table S1. [file JOA3-39-757-s001.docx]

**Supplemental Table 1. ICD therapy and VT/VF induction rates in patients undergone electrophysiologic study**

|  | Primary prevention (n = 64) | |  | Secondary prevention (n = 61) | |  |
| --- | --- | --- | --- | --- | --- | --- |
|  | Non-ischemic  (n = 47) | Ischemic  (n = 17) | P value | Non-ischemic  (n = 36) | Ischemic  (n = 25) | P value |
| VT/VF induction by EPS | 19 (40) | 10 (59) | 0.19 | 25 (69) | 17 (68) | 0.90 |
| Appropriate ICD therapy |  |  |  |  |  |  |
| VT/VF induction (+) | 6 (32) | 5(50)* |  | 11(44) | 3(18) |  |
| VT/VF induction (−) | 7 (25) | 0(0) |  | 2 (18) | 2 (25) |  |
| Inappropriate ICD therapy |  |  |  |  |  |  |
| VT/VF induction (+) | 3 (16)* | 0(0) |  | 1(4) | 2(12) |  |
| VT/VF induction (−) | 0 (0) | 0(0) |  | 0(0) | 1 (13) |  |

Values are number (%).

*P < 0.05 vs patients without VT/VF induction.

EPS: electrophysiologic study; ICD: implantable cardioverter-defibrillator; VT: ventricular tachycardia; VF: ventricular fibrillation; (+): induced by EPS; (−): not induced by EPS.
